# Assessment of left ventricular filling pressure using mean left atrial transit time from contrast enhanced dynamic MRI

**DOI:** 10.1186/1532-429X-13-S1-P20

**Published:** 2011-02-02

**Authors:** JJ Cao, Yi Wang, Jeannette McLaughlin, Elizabeth Haag, Michael Passick, Rena Toole, Joshua Y Cheng, Justine Lachman, Nathaniel Reichek

**Affiliations:** 1 St Francis Hospital, Roslyn, NY, USA

## Introduction

Left atrial (LA) size and function are often regarded as a reflection of left ventricular (LV) hemodynamics.

Purpose:In this study we investigated the hemodynamic correlates of contrast transit time within the left atrium (LA) in patients with LV systolic dysfunction by cardiac magnetic resonance imaging (CMR).

## Methods

Ten subjects undergoing clinically indicated right and left heart catheterization and 48 subjects undergoing noninvasive evaluation were prospectively enrolled and brain natriuretic peptide (BNP) and N-terminal proBNP (NT proBNP) obtained prior to CMR examination. CMR was performed within 5 hrs of invasive hemodynamic assessment. Dynamic MR imaging was acquired in sagittal and coronal planes covering the LA using a saturation recovery SSFP sequence with bolus injection of 0.01 mmol/kg gadopentetate. Mean transit time was measured in the LA and normalized to heart rate. LV systolic dysfunction was defined as LV ejection fraction (LVEF) < 50%. Noninvasive cohort also underwent echocardiography within 2 hours of CMR. Tissue Doppler was used to determine mitral E/e’ ratio.

## Results

Normalized mean LA transit time (nLATT) by CMR correlated strongly with LV early diastolic pressure (r=0.893, p=0.001), end diastolic pressure (LVEDP) (r=0.909, p<0.001) and mean diastolic pressure (r=0.936, p<0.001) in the invasive cohort. In the noninvasive cohort nLATT was significantly prolonged in patients with LV systolic dysfunction (N=39) 10.1±3.0 heart beats vs 6.6±0.7 heart beats in normal controls (N=9) (p<0.001). In patients with LV systolic dysfunction average LVEF was 37±9% and the NYHA functional class1.8±0.9. Using a linear regression equation derived from the invasive cohort LVEDP was estimated in the noninvasive cohort which was divided into 3 subgroups: ≤ 10 mmHg, 11-15 mmHg and ≥ 16 mmHg. There were graded increases from low to high LVEDP subgroups in echocardiographic mitral medial E/e’ ratio: 9±3, 14±7 and 18±13 (p=0.005); BNP: 53±41 pg/ml, 286±433 pg/ml, 496±475 pg/ml (p<0.001) and NT proBNP: 171±268 pg/ml, 712±948 pg/ml, 1249±1137 pg/ml (p<0.001), demonstrating the concordance of nLATT with established noninvasive indices of hemodynamic status.

## Conclusions

nLATT by dynamic MRI may be a valuable non-invasive marker of LV filling pressure in patients with LV systolic dysfunction.

